# Sleep and Aging. A Polysomnographic Follow‐Up Study, Some 40 Years Later

**DOI:** 10.1111/jsr.70039

**Published:** 2025-03-18

**Authors:** Peter Geisler, Renate Wehrle, Alexander Yassouridis, Alfred Ultsch, Thomas C. Wetter, Hartmut Schulz

**Affiliations:** ^1^ Center of Sleep Medicine, Department of Psychiatry and Psychotherapy University of Regensburg Regensburg Germany; ^2^ Ethics Committee Ludwigs‐Maximilians‐University Munich Germany; ^3^ Department of Informatics Philipps‐University Marburg Germany; ^4^ Department of Education and Psychology Free University Berlin Germany

**Keywords:** longitudinal study design, polysomnography (PSG), rapid eye movement density, REM sleep cycle, sleep stage transitions, sleep structure

## Abstract

The aim of the present study was to explore age‐related sleep alterations in normal subjects whose sleep had been recorded for the first time 40.4 ± 4.8 years ago. For the follow‐up polysomnography (3 nights) 15 participants (5 female, 10 male, age 56–74 years) were recruited. Recording conditions and sleep scoring were adapted to the previous study. In all older participants, the amount of slow‐wave sleep and REM sleep was decreased, while stage 1 and wakefulness were increased. There was no significant change in stage 2 or in any of the additional REM sleep parameters (REM density, latency, number and duration of REM cycles). Sleep stage transition analysis showed a markedly reduced stability of SWS from young to older age. While none of the single sleep parameters showed intra‐individual stability with age, a pattern analysis, which combined seven sleep parameters (sleep stages, total sleep time and REM density), showed that the concordance rate of the combined sleep parameters correlated significantly with the age at follow‐up. The results of this longitudinal study over a period of about 40 years are largely consistent with those of cross‐sectional studies. While the lack of significant correlation of the individual sleep parameters between the younger and older age groups did not allow for the identification of any of them as trait markers, the result of the pattern analysis, which combined a set of sleep parameters, indicates that the stability of the sleep structure decreases significantly in the age range between the late 50s and early 70s.

## 1 Introduction

Human sleep undergoes significant changes across the lifespan. Such changes have been the subject of intensive cross‐sectional studies from young adulthood to old age (Feinberg and Carlson 1968; Williams et al. 1974; Dijk et al. 2010). A comprehensive meta‐analysis of quantitative data on sleep structure from infancy to old age was conducted by Ohayon et al. (2004) and subsequently reviewed by several authors (Bliwise et al. 2005; Miner et al. 2022; Mander et al. 2017; Li et al. 2018; Campos‐Beltrán and Marshall 2021). According to these studies, an increase in sleep latency and involuntary awakenings during sleep, as well as a reduction of slow‐wave sleep (SWS) and a decrease of sleep spindles, represent key characteristics of sleep alterations with age. However, a comprehensive meta‐analysis (Boulos et al. 2019) employing the AASM scoring criteria (Iber et al. 2007) did not corroborate the reduction of SWS.

In contrast to these results from cross‐sectional studies, there is a paucity of knowledge regarding the intra‐individual alterations of polysomnographically measured sleep over a longer period of time in humans. The longest polysomnographic follow‐up currently available is that of Mokhlesi et al. (2014), which examined a large sample of patients with sleep apnea for 24 years with six follow‐up examinations. While follow‐up studies in healthy subjects have been conducted in children from school age to adolescence (Hedger‐Archbold et al. 2014), in young adults (Hahn et al. 2019) and in older adults (Carlson et al. 2008; Kawai et al. 2016), no such study has yet covered the time span from young adulthood to advanced age. For this reason, we took the chance to re‐examine the sleep structure again in a group of older individuals whose sleep had been recorded polysomnographically when they were young, several decades ago. In addition to re‐evaluating and expanding knowledge about global changes in sleep with age, the study should elucidate whether or not inter‐individual differences in the sleep parameters of young people are maintained over such a long period of time. To gain a more comprehensive understanding of the complex changes in sleep structure with age, the evaluation of the sleep data was extended beyond the standard sleep variables, such as sleep stages, latency measures and sleep cycle characteristics, to include analyses that integrate multiple parameters. For this purpose, a stage transition analysis and a pattern analysis were carried out as described in the Methods.

## 2 Methods

### 2.1 Study Design

The initial polysomnographic sleep recordings were conducted with a cohort of young, normal volunteers, aged 21–30 years, between the years 1975 and 1989. Each participant underwent a series of 14 to 30 consecutive nights of polysomnographic (PSG) sleep recording in the sleep laboratory of the Max Planck Institute of Psychiatry, Munich (Study 1). The results based on this data set have been published previously (Schulz et al. 1975, 1980; Wilde‐Frenz and Schulz 1983). The follow‐up study was conducted in the years 2021 and 2022 with three consecutive PSG recording nights per participant in the sleep laboratory of the Department of Psychiatry and Psychotherapy at the University of Regensburg (Study 2).

Those participants of Study 1 who were still available were contacted and invited to participate in Study 2. Sleep parameters from the initial three nights of Study 1 were compared with those from the three nights of Study 2. Prior to their participation in the study, all participants provided informed consent to the study procedure and to the use of their data from Study 1 for comparison with data from Study 2. The study was approved by the ethics committee of the University Clinic of Regensburg.

### 2.2 Polysomnographic Recording and Sleep Scoring

In Study 1, electrode placement and recording conditions adhered to the rules of the Rechtschaffen and Kales manual (Rechtschaffen and Kales 1968). In Study 2, the recordings were conducted following the standards set forth in the AASM manual, version 2.6 (Berry et al. 2020) for attended full cardiorespiratory video‐polysomnography. This comprised an electroencephalogram (EEG) (6 channels), an electrooculogram (EOG) (2), an electromyogram of the chin (chin EMG) (2), oral and nasal airflow, respiratory effort (thoracic and abdominal), snoring sound, transcutaneous oxygen saturation, an ECG, heart rate, tibialis anterior EMG bilaterally and body position (night 1). To minimise discomfort for the participants and to align the recording conditions with those of Study 1, the respiratory and leg EMG sensors were not applied during nights 2 and 3. The recordings were conducted with a Nihon‐Kohden Neurofax 32‐channel PSG system (Version 6.5) in conjunction with a Polysmith PSG analysis software (Version 11).

The lights were turned off at the discretion of the participants at 23:00 +/− 1 h, and recording was stopped after 8 h (960 epochs). Participants were instructed to remain in bed until the designated end of the recording, except for toilet visits, even if they were awake earlier. All participants were instructed to maintain their current medication regimens and, in two cases, CPAP treatment throughout Study 2.

To prevent any unintended effects that might result from differences in recording duration between Study 1 and Study 2, the corresponding nights from the two studies were trimmed to the same length, thereby ensuring an equivalent time in bed (TIB) for the paired recordings.

In Study 1, sleep stage scoring according to the R&K criteria was conducted by two experienced scorers, and any cases requiring further scrutiny were discussed with a senior sleep researcher. Moreover, rapid eye movements in REM sleep were analysed using three‐second mini‐epochs (see below). The PSG raw data from Study 1, which were stored on analog tape originally, were not archived any more, while the results of the 30‐s epoch visual sleep stage scoring and the rapid sleep eye movement density data from Study 1 were still available in digital form for all participants.

The sleep data from Study 2 were scored twice, initially in accordance with the AASM manual version 2.6 and subsequently in strict adherence to the R&K manual. For the R&K scoring, the displayed channels, filters and amplifications were modified in such a way that the montage was in full conformity with the R&K requirements. This permitted a direct comparison of the PSG data from both studies. All comparisons between studies are based on the R&K analysis. For further analysis, stages S3 and S4 were combined and will be reported as slow‐wave sleep (SWS). The PSG recordings from Study 2 were independently scored by two experienced scorers. Their primary concordance rate was greater than 90%. Any discrepancies were discussed, and remaining disagreements or unusual findings were clarified with a senior sleep specialist (PG, RW). The scorers of Study 2 were blinded to the scoring results of Study 1.

### 2.3 Rapid Eye Movement Sleep Analysis

Rapid eye movements were rated visually in both studies. Each 30‐s epoch of REM sleep was segmented into 10 3‐s mini‐epochs. The number of mini‐epochs containing at least one rapid eye movement was counted, resulting in a value between zero and 10 per REM sleep epoch. The mean REM eye movement density (RD) was calculated as the arithmetic mean of this score per epoch of actual REM sleep.

In the case of nights comprising a minimum of three complete REM periods, the RD of the initial three REM periods was compared according to their sequential position. As the RD was evaluated in the two studies by different scorers, and a gold standard for the evaluation of this parameter does not exist, the non‐parametric Friedman test for repeated measures was employed for statistical analysis.

The termination of a REM period was defined as the final epoch scored as REM sleep that was not followed by another epoch of REM sleep within a 20‐min interval. Any instances of REM sleep interruptions by wake or NREM sleep (also known as “intrusions”) were incorporated into the overall duration of the REM period. REM sleep efficiency was defined as the percentage of actual REM sleep epochs within a given REM period. The final REM period of a PSG record was considered incomplete when the recording terminated within 20 min after the final REM epoch. Incomplete REM periods were excluded from the analysis of REM periods.

In order to characterise the cyclic structure of sleep, REM cycles were considered. The duration of a REM cycle was defined as the temporal distance between the first epoch of two consecutive REM periods, as suggested by Williams et al. (1974). A REM cycle was classified as complete when it was terminated by the onset of the subsequent REM period, irrespective of whether this subsequent REM period was complete or incomplete. To ensure homogeneity, the statistical analysis was limited to the initial four REM periods and REM cycles of each night.

### 2.4 Study Participants and Procedures

The index cards of the original study participants were still available. This enabled the identification of 19 of the participants of Study 1. A great number of those who participated in the follow‐up study (Study 2) were still in contact with one or more of the present investigators. Of the 15 individuals who were successfully contacted, all agreed to participate in Study 2. With regard to the remaining four individuals, one had passed away, in two cases contact data were unavailable, and one did not respond to our request. The mean interval between the two studies was 40.5 years (SD 4.8 years, range 33–46 years). The mean age of the participants in Study 1 was 25.4 years (SD 2.6 years, range 21–30 years), while that of participants in Study 2 was 65.9 years (SD 5.4 years, range 58–75 years). A summary of the biographical and current sleep‐related clinical data of the participants is presented in Table 1. No explicit exclusion criteria were applied for Study 2 in order to obtain a sample as unbiased as possible.

**TABLE 1:** Demographic and clinical data of participants.

Participant ID	Sex	Age (yrs) at Study 1	Age (yrs) at Study 2	Age diff.	BMI at Study 2^a^	Documented medical conditions at Study 2	Sleep related treatment/medication	AHI	PLMAI
SW01	f	29	74	46	19			5	1
SW02	m	24	70	46	n.a.			0	51
SW03	m	28	74	46	26			10	6
SW04	m	25	65	40	21			1	1
SW05	m	25	58	33	21			8	0
SW06	f	22	68	46	18			3	4
SW07	f	28	66	38	23	AH		5	14
SW08	m	26	66	40	31	AH, CHD, Diab. mell.		17	2
SW09	m	26	65	39	29			29	0
SW10	m	26	71	45	24			1	2
SW11	f	24	67	43	32			2	0
SW12	m	23	56	33	28	OSA	nAPAP (5–15 mbar)	0	6
SW13	m	21	60	39	27	HC		11	4
SW14	f	30	63	33	26			n.a.	n.a.
SW15	m	24	64	40	31	HC, OSA	nAPAP (8–13 mbar)	3	0
Mean		25.4	65.8	40.4	25.4				
SD		2.6	5.3	4.8	4.5				

*Note*: Basic demographic and clinical data of the study participants, as assessed at Study 2.

Abbreviations: Age diff = difference between age at Study 1 and Study 2; BMI = body mass index; AH = arterial hypertension, treated, CHD = coronary heart disease, Diab. mell. = diabetes mellitus, type 2, HC = hypercholesterinemia, OSA = obstructive sleep apnea, nAPAP = nasal continuous positive airway pressure, autoadjusted, with pressure range, AHI = Apnea‐Hypopnea Index (events/h of sleep), PLMAI = periodic leg movement related arousal index (events/h of sleep), n.a. = not available.

^a^ BMI at Study 1 not available.

The 15 available participants of Study 1 were fully informed of the conditions of the follow‐up study, and they were invited for three consecutive nights of PSG recording in the sleep laboratory of the Psychiatric Clinic, Regensburg. Prior to their admission to the sleep laboratory, the participants were provided with the following questionnaires: BDI (Hautzinger et al. 1995), D‐MEQ (Griefahn et al. 2001), MCTQ (Roenneberg et al. 2003), ESS (Johns 1991), ISI (Bastien et al. 2001), PSQI (Buysse et al. 1989), IRLS Rating Scale (Trenkwalder et al. 2001), RIS (Crönlein et al. 2013) and a 14‐day sleep diary to complete and bring with them on admission to the sleep unit. They were asked to maintain a regular sleep–wake schedule throughout the duration of the study and to avoid shift work and flights across multiple time zones.

The participants arrived at the clinic in the afternoon preceding the first night's sleep recording. Upon arrival, a brief medical interview and physical examination were conducted to rule out any acute medical conditions and to confirm the eligibility of the participants in accordance with the established inclusion/exclusion criteria. In the days between the sleep recordings, the study participants were free to dispose of their time at their discretion. The recording room, which was set up like a hotel room, complete with bathroom facilities, was accessible for them throughout their stay, and meals were supplied. Participants were reimbursed for their travel expenses and given an allowance for their time and effort.

### 2.5 Deviations From the Study Plan

Three participants were unable to present themselves at the sleep laboratory for data acquisition due to personal circumstances. In two instances (SW10, SW11), home recordings were conducted with a mobile recording system of the same type as the one used in the sleep laboratory for three consecutive nights, with settings comparable to those utilised for in‐house sleep recordings. The home recordings were conducted and monitored continuously from an adjacent room during the night by one of the authors (RW). In one instance (SW14), the PSG recordings were conducted with the assistance of a collaborating sleep clinic in Vienna, Austria. The same standards were applied as those used at the Regensburg laboratory. In two other cases, the series of three‐night sleep recordings had to be repeated due to a technical issue on the second night (SW04) and due to an irregular sleep schedule prior to the PSGs (SW09). The latter participant consented to adhere to a more regular sleep regimen at home, which was monitored by a two‐week continuous actigraphic home recording. Following this, a second series of three PSGs was conducted. In these two cases, the second series of sleep recordings was used as the basis for analysis.

During the acute phase of the SARS‐CoV‐2 pandemic, all study‐related sleep recordings were temporarily halted. Upon the resumption of the recordings in 2021, the clinic's established hygiene protocols were rigorously adhered to. Recordings were postponed when participants, in consideration of their advanced age, felt it was not safe to travel. Consequently, the initial polysomnography (PSG) was conducted in August 2021, and the final participant was recorded in November 2022.

### 2.6 Data Analysis

The influence of the factors ‘age group’ and ‘night repeat’ on the sleep stages S1, S2, SWS (S3 + S4), REM and WAKE was tested with Wilks multivariate tests of significance in a multivariate analysis of variance with a repeated measures design on the basis of the sample of all nights (*n* = 90) from the two studies.

To eliminate the effect of different recording durations, all sleep stage variables were referenced to TIB for statistical analysis and expressed as percentages thereof (W%, S1%, S2%, SWS%, REM%). For other parameters of interest (sleep latencies, duration of REM sleep periods and REM cycles), absolute values (in minutes) were employed. The mean values for each sleep parameter were calculated for the three corresponding nights. The differences between the mean values from Study 1 and Study 2 were tested for statistical significance using the paired Wilcoxon signed‐rank test. To evaluate the magnitude of change, Cohen's d was calculated. To determine whether the value of any parameter at young age was correlated with the respective value at older age, Spearman's rank correlations were calculated. Missing data are indicated where applicable. For comparisons, a significance level of 0.05 was applied. Since the evaluation of the study is to be understood much more on the exploratory and less on the inferential level, no correction of the significance level should be necessary for multiple testing, and no Bonferroni corrections were applied. This should be observed in the interpretation of the results.

The building blocks of a sleep profile are the sleep stages, which are defined as successive series of epochs of the same stage. The objective of the contingency analysis was to determine the transition probabilities within and between the stages W, S1, S2, SWS and REM. For each single epoch, the consecutive epoch was considered. When the consecutive epoch was of the same stage as the previous one, this was a within‐stage transition; else, a transition to the respective stage was counted. The probability of these different transitions, relative to all transitions originating from this stage, was tabulated as a percentage value in a transition matrix. The contingency of the stages was determined by calculating the frequency of the within‐stage transitions separately for the young and the older participants. High percentages of within‐stage transitions indicate stable sleep stages (contingency). Stage contingency was defined as a within‐stage transition probability of > 50%. Each group comprised 45 sleep recordings.

The individual trajectories of the sleep parameters of interest were analysed to determine whether and to what extent a consistent pattern of directional change occurred in the transition from young to older age. At the individual level, the quantitative changes were classified as either an increase or a decrease for each parameter, with the former represented by a positive rank (‘+’) and the latter by a negative rank (‘−’). The correlation of individual scores between Study 1 and Study 2 was evaluated using Spearman's correlation coefficients.

In order to evaluate the degree of similarity or dissimilarity between the sleep patterns of young and older individuals, a pattern analysis was performed. For this analysis, the following seven sleep variables were selected: TST, W%, S1%, S2%, SWS%, REM% and RD. Individual values at or above the group median were indicated by a plus sign (+), while those below the median were indicated by a minus sign (−). This yielded seven pairs of combinations of plus and minus signs for each participant. A high number of congruent pairs (++ or −−) indicates greater similarity between the sleep patterns of young and older ages, while a low number of congruent pairs indicates a lack of similarity. Spearman's rank correlation coefficients were computed between the number of congruent pairs and the time difference between Study 1 and Study 2, as well as the age at Study 2.

Study methods and results are reported following the Strengthening the Reporting of Observational Studies in Epidemiology (STROBE) Statement for cross‐sectional studies (von Elm et al. 2007). The data underlying this article, which do not allow the identification of participants, will be shared on reasonable request to the corresponding author.

## 3 Results

### 3.1 Study Participants

Participants (10 male, 5 female) ranged in age from 21 to 30 years in Study 1 and from 56 to 74 years in Study 2, with a mean interval of 40.4 years between the two sets of sleep recordings. For Study 1, mostly students were recruited. None of the participants reported any medical conditions or sleep‐related medications at the time of Study 1. At the time of Study 2, all participants lived independently in their homes. Nine of them lived with their partners; the others lived alone. Five participants indicated that they worked full‐time, one worked part‐time, and nine were retired. Two men had known obstructive sleep apnea and were adequately treated with CPAP. Biographical data, medical conditions, and treatments at the time of Study 2 are listed in Table 1.

### 3.2 Medical Findings and Questionnaires

The medical examination conducted at the time of admission revealed no conditions that could potentially interfere with the conduct of the study. All participants were in good general physical and mental condition. In the polysomnographic recordings from night 1 of Study 2, two participants (SW08, SW09) exhibited a previously undocumented clinically relevant apnea‐hypopnea index without associated symptoms. One participant (SW02) showed a high index of periodic leg movements in association with arousals, without any reported symptoms of a restless legs syndrome (IRLS score: 0) or subjectively fragmented sleep (PSQI overall sleep quality score 1 ≙ ‘fairly good’). The participants were informed of these findings and advised to contact their family physicians regarding them. One of the older participants (SW03) had unusually short REM sleep latencies on all three nights (11.5, 17 and 13 min), which qualified as sleep onset REM periods. However, no symptoms of narcolepsy or excessive daytime sleepiness were observed (ESS score: 9). The questionnaire data collected at the time of Study 2 did not indicate the presence of any clinically relevant sleep‐related problems, apart from mild to moderate difficulties with sleep initiation and maintenance (*n* = 3), elevated daytime sleepiness (*n* = 2) and mild RLS symptomatology (*n* = 1). The scores of the applied questionnaires are displayed in Table S1; the interpretation of these scores is outlined in Table S2.

### 3.3 Sleep Stages

A multivariate analysis of the factors ‘age group’ and ‘night repeat’ revealed a significant global effect of age on the duration of W, S1, S2, SWS and REM with a power of approximately 100% (effect of ‘age group’ → *F* (5,10) = 26.7, *p* < 0.001). However, no significant effect was observed for the factor ‘night repeat’ or for its interaction with age. Therefore, the mean values of the PSG data sets for each sleep variable were pooled for further analyses, unless otherwise stated.

**FIGURE 1:**
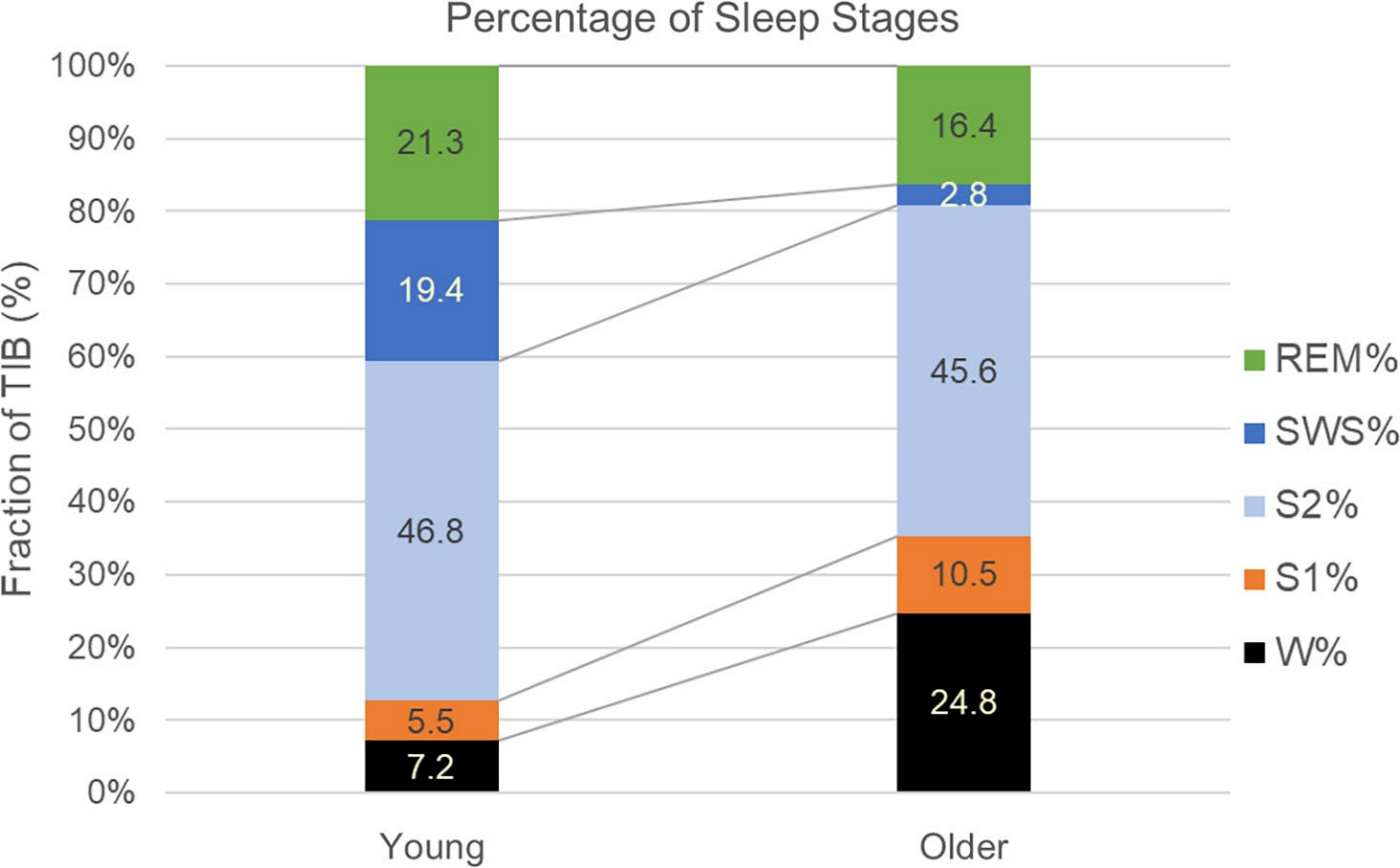
Sleep stage percentages for the young and older participants, referenced to Time in Bed. The duration of all stages expressed as percentage of Time in Bed shows a marked increase of Wake and Stage 1 sleep, while Slow wave sleep (stages S3 and S4) decreases strongly, and REM sleep decreases to a lesser, but significant extent. W%: Stage Wake; S1%: Sleep stage 1; S2%: Sleep stage 2; SWS%: Slow wave sleep (stage 3 + stage 4); REM%: Sleep stage REM. Young: Mean values from Study 1; Older: Mean values from Study 2. The differences between the Young and the Older age groups are significant for W%, S1%, REM% and SWS%. (paired Wilcoxon rank test, *p* < 0.01, *n* = 15).

The percentages of sleep stages, expressed as a percentage of TIB (W%, S1%, S2%, SWS%, REM%) exhibited notable differences between the young and the older participants (Figure 1). SWS% decreased from 19.4% ± 6.5% to 2.8% ± 3.5% (paired Wilcoxon signed‐rank test, *p* < 0.001, Cohen's *d* = −2.4) and REM% decreased from 21.3% ± 4.2% to 16.4% ± 4.1% (*p* = 0.006, *d* = −0.97). Conversely, W% increased from 7.2% ± 3.2% to 24.8% ± 11.3% (*p* < 0.001, *d* = 1.6), and S1% increased from 5.5% ± 2.0% to 10.5% ± 4.0% (*p* < 0.001, *d* = 1.3). The change of S2 was not significant. The latency measures (SOL (S1), SOL (S2), REM latency) did not change significantly with age group either (Table 2).

**TABLE 2:** Comparison of polysomnographic sleep parameters at young (Study 1) and older age (Study 2), referenced to time in bed (TIB).

	Study 1 (young)	Study 2 (older)	Wilcoxon test^e^	Effect size
	Mean ± SD	Mean ± SD	*p*, 2‐sided	Cohen's d
TIB (min)	464 ± 13.8		—	—
Wake %	7.2 ± 3.2	24.8 ± 11.3	< 0.001	1.6
S1%	5.5 ± 2.0	10.5 ± 4.0	< 0.001	1.3
S2%	46.8 ± 5.3	45.6 ± 9.4	0.69	−0.10
SWS %	19.4 ± 6.5	2.8 ± 3.5	< 0.001	−2.4
REM %	21.3 ± 4.2	16.4 ± 4.1	0.006	−0.97
SOL (S1) (min)	13.1 ± 8.1	15.9 ± 9.5	0.48	0.19
SOL (S2) (min)	18.6 ± 8.8	19.6 ± 11.4	0.69	0.06
REM‐latency (min)^a^	91.8 ± 32.2	75.2 ± 39.0	0.16	−0.38
REM density (0–10)^b^	2.7 ± 0.8	3.0 ± 0.4	0.26	0.30
REM period count (*n*/night)	3.4 ± 0.5	3.2 ± 0.7	0.67	−0.20
REM period duration (min)^c^	25.4 ± 14.4	21.3 ± 12.5	0.07	−0.53
REM cycle count (*n*/night)	3.2 ± 0.6	3.0 ± 0.8	0.27	−0.29
REM cycle duration (min)^d^	95.7 ± 7.7	107.7±	0.10	0.51
Awakenings^f^/h	0.86 ± 0.38	4.1 ± 1.4	< 0.001	3.21
Stage changes/h	14.2 ± 3.9	17.6 ± 4.3	0.06	−0.89

*Note*: Polysomnographic sleep parameters, compared between Study 1 and Study 2, based on the mean values from three nights for each individual (*n* = 15). Differences between both groups were evaluated nonparametrically by the Wilcoxon rank test, the size of the difference is expressed as Cohens d. TIB = Time in bed, trimmed to the duration of the shorter one of each pair of corresponding nights (young/older); SOL (S1) = time from lights out to first epoch of S1 or any other sleep stage (sleep onset latency); SOL (S2) = Time from lights out to first epoch of S2; REM‐Latency = time from first epoch of any sleep stage to first epoch of REM sleep (REM‐latency).

^a^ One night with a single REM‐cycle was excluded (SW06, older, night 1).

^b^ *n* = 14 due to missing data in one young subject.

^c^ Calculation of REM cycle duration limited to completed REM periods and REM cycles 1–4.

^d^ Nights with less than 2 REM cycles were excluded, calculation limited to REM cycles 1–4.

^e^ Paired Wilcoxon signed rank test.

^f^ Awakening: Stage change from any sleep stage to stage wake.

In an exploratory analysis, the data from the two participants with untreated sleep apnea and the one participant with significant sleep disruption due to a high amount of PLMS were excluded. Excluding these three participants, the percentage of Wake decreases from 24.8% to 23.4%, and the percentage of S1 decreases from 10.5% to 9.8%. The level of significance for differences between Study 1 and Study 2 for all variables reported in Table 2 remained unchanged. Therefore, the full sample was kept for further analysis.

When sleep stages were scored according to the AASM criteria with the AASM standard EEG montage in the older participants, the percentage of stage N3 was significantly higher than the SWS% in the R&K scoring, with a percentage of 9.8% ± 6.6% (paired Wilcoxon signed rank test, *p* < 0.001) (Table S3). However, this percentage was still significantly lower than the SWS% value scored according to R&K when the participants were young (Wilcoxon test, *p* < 0.001).

### 3.4 Sleep Stage Transitions (Contingency Analysis)

**FIGURE 2:**
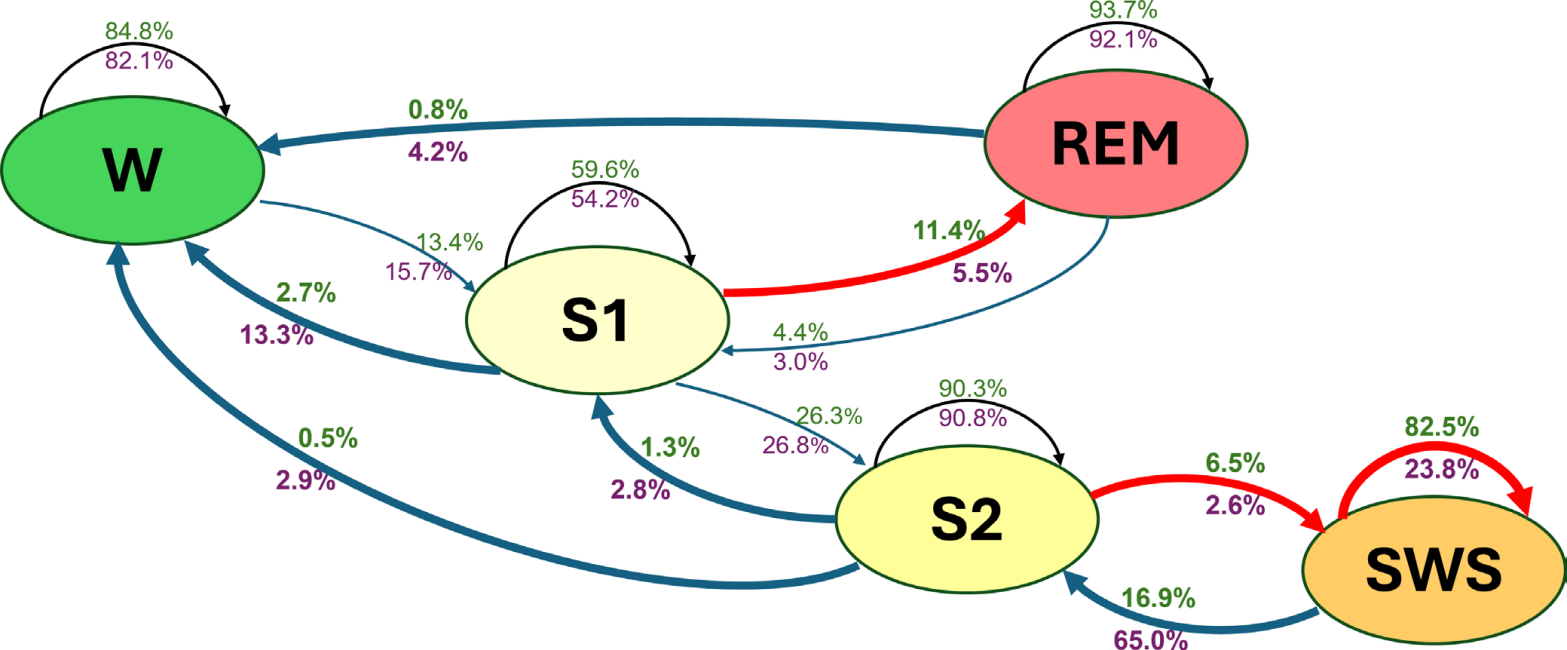
Sleep stage transition probabilities for young and older participants (Contingency analysis). Transition probabilities in percent of successive epochs within and between sleep stages for the young (green numbers) and the older age group (magenta numbers). Higher values for within‐stage transition probabilities (arrows begin and end within the same box) indicate longer episodes of stable sleep stages. Increases of transition probabilities from the young to the older age group are indicated by blue, and decreases by red arrows. Significant changes (*p* < 0.05) are highlighted by bold arrows. The distance of the boxes is arbitrary. For clarity, probabilities less than 1.5%, that were not significantly different between age groups, are not shown. The complete transition matrix is given in Table S4.

In the young participants, all sleep stages exhibited contingent transition patterns, defined as a within‐stage transition probability greater than 50%. These probabilities ranged from 93.7% for REM sleep to 59.6% for S1. With the exception of SWS, all sleep stages remained contingent at older age. The contingency value of SWS exhibited a pronounced decline, decreasing from 82.5% in the young participants to 23.8% in older age. Conversely, the transition from SWS to S2 increased from 16.9% in young age to 65% in older age. In contrast, the transition from S2 to SWS decreased by two‐thirds from young to older age (6.5% to 2.6%). Figure 2 illustrates the contingency values and transitions for the young and the older participants. For the complete transition matrix see Table S4.

Several other between‐state transitions changed substantially from young to older age. The transition from S1 to REM was reduced by half (11.4% vs. 5.5%), while the transition from S1 to W was increased fivefold (2.7% vs. 13.3%). The transition probability from REM to W increased from young to older age from 0.8% to 4.2%. In total, the rate of transitions to stage W (awakenings) increased from 0.86 ± 0.38 to 4.1 ± 1.4 per hour of TIB (Wilcoxon signed rank test, *p* < 0.001, Cohen's *d* = 3.29). The total rate of stage changes increased moderately from 14.2 ± 3.9 to 17.6 ± 4.3 per hour of TIB, approaching significance (*p* = 0.06) (Table 2).

### 3.5 REM Sleep Analysis

The total number of completed REM periods (see Methods) was 158 in the young and 154 in the older participants (3.5 ± 0.6 vs. 3.4 ± 0.9 REM periods per night). Five REM periods occurred in just 4 nights in the young and in 6 nights in the older participants, whereas 6 REM periods in one night were recorded once in one older participant (SW03). Restricting the analysis to the first four REM periods of each night (see Methods) resulted in 154 REM periods for analysis in the young group and 146 in the older group. The mean duration of completed REM periods was slightly longer in the young than in the older participants (25.4 ± 14.4 vs. 21.3 ± 12.5 min), but the two groups did not differ significantly (*n* = 2 × 15, Wilcoxon test n.s., Table 2). REM sleep efficiency was similar in both age groups (young: 89.4% ± 5.0%, older: 87.7% ± 5.0%, paired Wilcoxon test n.s.). However, the composition of the intrusions into REM periods was clearly different. In the young group, the intrusions consisted of 13% stage W and 87% NREM, while in the older individuals, the ratio was 39% W and 61% NREM.

The majority of nights had either 2 (16%), 3 (51%) or 4 (24%) complete REM cycles, while 5 or 6 cycles (4%) and less than 2 cycles (4%) were rare. The mean number of REM cycles per night was similar in both age groups (3.2 ± 0.6 vs. 3.0 ± 0.8, n.s.), but the extremes (none or 6 cycles) occurred only in the older participants (Table S5). The statistical analysis was limited to REM cycles 1 to 4. Three nights with less than 2 cycles were excluded to avoid bias due to irregular nights, leaving a total of 43 nights with 144 completed REM cycles in the young and 42 nights with 127 completed cycles in the older participants for analysis. The mean duration of the REM cycles was approximately 10 min longer, and the standard deviation was larger in the older than in the young participants, but the difference was not significant (young: 95.7 ± 7.7 min, older: 107.7 ± 20.9 min, paired Wilcoxon test n.s., *p* = 0.053). In a univariate ANOVA, the sequential position of the REM cycles within the night had a significant effect on cycle duration (ANOVA, *p* = 0.001). Age group, sequential position of the night within the study, and the interaction terms did not show any significant effects. REM cycle 1 was the shortest (99.6 min, 95% CI 93.5–105.8) and REM cycle 2 the longest one (108.0 min, 95% CI 101.9–114.2).

Phasic eye movement activity expressed as REM density (RD, calculated as mean per participant) did not differ with age (young: 3.0 ± 0.4, older: 2.7 ± 0.8, paired Wilcoxon test, n.s., Table 2). The correlation between the RD values from Study 1 and Study 2 for the individual participants was low (Spearman's rho = 0.12, n.s., Table 3).

**TABLE 3:** Intraindividual variations and correlations of sleep parameters between Study 1 and Study 2.

	(−) ranks	(+) ranks	*p* (Sign test)	Correlation (Spearman's rho)	*p* (correlation)
Wake %	0	15	< 0.001	0.31	0.27
S1%	0	15	< 0.001	0.25	0.36
S2%	6	9	0.61	−0.13	0.65
SWS %	15	0	< 0.001	0.50	0.06
REM %	12	3	0.04	0.24	0.38
SOL (S1)	7	8	1.00	−0.51	0.05
SOL (S2)	7	8	1.00	−0.47	0.08
REM‐latency^a^	10	5	0.30	0.26	0.35
REM density^b^	5	9	0.42	0.00	0.99
REM periods/night	8	7	1.00	−0.18	0.52
Mean REM period duration^c^	10	5	0.30	0.12	0.66
REM cycles/night	8	4	0.39	−0.05	0.86
Mean REM cycle duration^a^, ^c^	5	10	0.30	−0.10	0.73
Awakenings^d^/h TIB	0	15	< 0.001	0.47	0.08
Stage changes/h TIB	5	10	0.30	−0.02	0.95

*Note*: Mean values of polysomnographic variables for night 1–3 from both studies for each participant were used (*n* = 15). Differences between Study 1 and Study 2 were tested by sign test. The correlation between the mean values from Study 1 and Study 2 was calculated as Spearman's rho; the significance level of the correlation is indicated in the last column. All correlations were non‐significant. For abbreviations see Table 2.

^a^ One night with a single REM‐cycle was excluded (SW06, older, night 1).

^b^ *n* = 14 due to missing data in one young subject, else *n* = 15.

^c^ Calculation limited to REM periods/REM cycles 1–4.

^d^ Awakening: Stage change from any sleep stage to stage wake.

The analysis of RD according to their sequential position within the night showed a significant effect for both age groups (Friedman's test for repeated measures, young: *X* ^2^_r_ = 30.2 (df = 2, *n* = 39), *p* < 0.001; older: *X* ^2^_r_ = 10.9 (df = 2, *n* = 40), *p* = 0.004), with the first REM period being the one with the lowest RD (Table S6).

### 3.6 Intraindividual Changes

**FIGURE 3:**
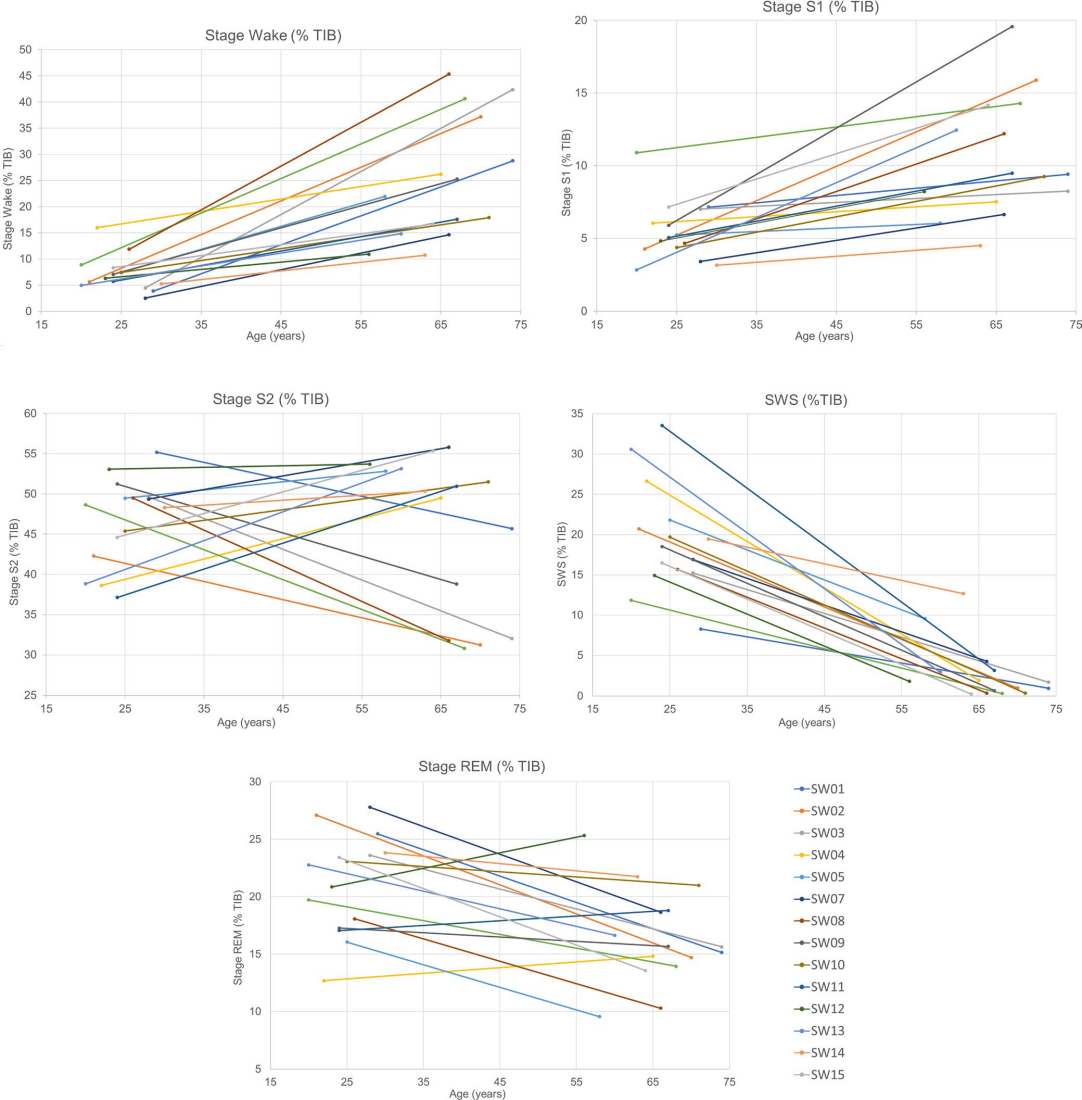
Line plot of the changes in individual sleep stage percentages for the young (Study 1) and older (Study 2) participants. Individual percentages of sleep stages, referenced to time in bed (TIB), from young (21–30 years) to older (56–74 years). The lines connect the mean values of the three nights of Study 1 and Study 2 for each individual. On the *x*‐axis: Age of participants at Study 1 and Study 2 respectively.

Comparing the individual results of young and older participants, the changes in sleep stages showed basically the same pattern as the global parameters in each of the 15 participants, with an increase in W% and S1%, a steep decrease in SWS%, and a moderate decrease of REM%, while S2% showed no uniform pattern, as visualised in Figure 3. The latency measures (SOL (S1), SOL (S2)) and the REM sleep parameters did not show uniform trends either (Table 3). Spearman's correlation coefficients between the values of the young and the older age groups for each variable were low and not significant for all variables tested (Table 3). Only a small fraction (22%–0.5%) of the variability is explained by the correlation with age.

### 3.7 Pattern Analysis of Sleep Parameters

To examine the stability of sleep components over time, the position of a given participant relative to the group median in Study 1 vs. Study 2 was determined for 7 sleep parameters (Methods). The number of congruent pairs varied between 7 (SW14) and 1 (SW02, SW03). The rate of congruent signs was negatively correlated with the age of the participants at Study 2 (Pearson's *r* = 0.67, *p* = 0.006) and with the interval between the two studies (Pearson's *r* = −0.63, *p* = 0.01). Age at Study 2 and the interval between the two studies were closely correlated (*r* = 0.82, *p* < 0.001). The result indicates a decrease in intra‐individual stability of the sleep pattern with increasing age (Table 4).

**TABLE 4:** Median split pattern analysis of differences between Study 1 and Study 2 for selected polysomnographic parameters.

ID	Age; young, older	Age diff.	TST min	W %	S1%	S2%	SWS %	REM %	RD	Congruent pairs
SW14	30, 63	33	++	−−	−−	++	++	++	++	7
SW05	25, 58	33	++	++	+−	++	++	−−	++	6
SW06	22, 68	46	−−	++	++	+−	−−	−−	n.a.	5
SW13	21, 60	39	++	−−	−+	−+	++	++	−−	5
SW04	25, 65	40	−−	++	+−	−−	++	−−	−+	5
SW07	28, 66	38	−+	−−	−−	−+	−+	++	−−	4
SW12	23, 56	33	++	+−	−−	++	−+	−+	−−	4
SW08	26, 66	40	−−	++	−+	+−	−−	−−	+−	4
SW09	26, 65	39	−−	++	++	+−	+−	−+	++	4
SW11	24, 67	43	++	−−	++	−+	++	−+	+−	4
SW15	24, 64	40	−+	+−	++	−+	−−	+−	++	3
SW01	29, 74	46	+−	−+	++	+−	−−	+−	−−	3
SW10	26, 71	45	−+	+−	−−	−+	+−	++	+−	2
SW02	24, 70	46	+−	−+	−+	−−	+−	+−	−+	1
SW03	28, 74	46	+−	−+	+−	+−	−+	++	−+	1

*Note*: Age at Study 2 versus number of congruent pairs: rho = −0.67, *p* (2‐tailed) = 0.006. Age difference versus number of congruent pairs: rho = −0.64, *p* (2‐tailed) = 0.01. Pattern of the position of the individual study participants at the two study time points (young, Study 1/older, Study 2), in relation to the group median of the respective sleep variables. Plus sign: value at or above the group median. Minus sign: value below the group median. Pairs are congruent (highlighted in grey), when both signs in a box are the same. The number of congruent pairs in each individual decreases significantly both with age at Study 2 and, to a lesser degree, with the time lapse between Study 1 and Study 2, expressed as age difference. W%, S1%, S2%, SWS%, REM%: Percentage of the respective sleep stages referenced to TIB; RD: REM density. rho: Spearman's rank correlation coefficient. Age diff. = difference between age at Study 1 and Study 2 (years).

## 4 Discussion

The findings of the present longitudinal study are largely consistent with those of cross‐sectional studies conducted on people within the age range from young adulthood up to the seventh decade of life (Feinberg and Carlson 1968; Williams et al. 1974; Spiegel 1981; Lauer et al. 1991). The primary alterations in sleep structure with advancing age, as reported in these studies, are a prolonged sleep onset latency, an increase in sleep interruptions, and a reduction in slow‐wave sleep. These factors contribute to impaired sleep maintenance and reduced sleep efficiency in older individuals. The majority of these typical age‐related alterations in sleep structure were corroborated in the present longitudinal study. As subjects progressed from young to an older age, there was a significant increase in the amount of time awake and stage 1 sleep, accompanied by a significant reduction in SWS (S3 + S4) and REM sleep. These results are similar to those of Lauer et al. (1991), who reported a decline of SWS% from 16.6% in their healthy controls aged 25–34 years to 5.0% in the 55–65‐year‐olds, and a decrease of sleep efficiency from 93.0% to 81.6% in the same groups. The percentile reference charts for 20‐ to 80‐year‐old healthy subjects from the SIESTA database show an increase in W and S1, and a decrease in S2 and REM sleep in the age range from 50 to 70 years (Danker‐Hopfe et al. 2005), which is consistent with the present results.

The finding of a prolonged sleep onset latency (e.g., Ohayon et al. 2004; Mander et al. 2017) with advanced age was not replicated in our sample (Table 2), which may be due to inadvertent differences in the setting of the sleep recording conditions between Study 1 and Study 2, or by insufficient statistical power to detect such a difference.

Another noteworthy aspect of the present results is the observed instability of the sleep process. In the older participants, sleep interruptions by wake episodes and by episodes of light sleep (S1) were more frequent. The contingency analysis (Figure 2) showed a prominent instability of SWS in the group of older subjects. Within‐stage recurrence of SWS was much reduced from 82.5% to 26.1% from young to older age, and the transitions from stage 1, stage 2, and stage REM to stage W were clearly increased in the older participants. The SWS findings are consistent with those of Park et al. (2022). Other state transition scores are difficult to compare between the two studies, because our analysis is based on the comparison of the same individuals at two different ages, whereas Park et al. had used a cross‐sectional design involving two independent age groups. Taken together, these results are consistent with the concept of functional uncertainty of sleep. This is a characteristic feature of sleep in early development (Salzarulo et al. 1997) and in older age (Conte et al. 2014). Interestingly, the cyclic structure of sleep may be less susceptible to this phenomenon as evidenced by the findings of unaltered REM period and REM cycle durations in the older age group.

Rapid eye movement density (RD) was the only phasic sleep parameter available for analysis in the present study. While Darchia et al. (2003, 2004), who had used digital oculogram data, observed a decrease of RD with age, this was not the case in the present study with visually scored eye movement data. This is again in line with the findings of Lauer et al. (1991), who also used visual eye movement analysis, in the above‐mentioned age groups. However, the comparison of RD data between Study 1 and Study 2 based on visual analysis may not be completely reliable due to the lack of standard scoring criteria, and an EOG‐based automated quantitative analysis, as in the studies of Darchia et al., was not possible. However, the sequential effect of a significantly lower RD score in the first REM period of the night was confirmed for both young and older participants in the present study. This finding is consistent with the results of two previous studies with visual RD scoring (Geisler et al. 1987; Peters et al. 2014) while quantitative studies concluded that RD in the first REM period was low in young but not in older normal individuals (Darchia et al. 2003, 2004).

A key objective of our study was to investigate whether there is evidence of long‐term stability of sleep parameters from young adulthood to older age. None of the analysed parameters of sleep macrostructure showed a significant correlation of the values from young to older age. Spearman's correlation coefficients ranged from −0.51 (Sleep Onset Latency) to 0.47 (Awakenings/h of TIB), and none of them were significant. Therefore, no single sleep parameter could be identified as a trait marker. However, when a set of sleep parameters was combined in a pattern analysis, a close correlation with age became apparent (Table 4). While the congruence between the original and the follow‐up pattern was high for the younger members of the older participant group in our sample, it decreased with increasing age at follow‐up. This suggests that the age period between the late 50s and the early 70s may be a crucial period regarding the changing sleep structure in older individuals. Factors that may contribute to this decrease in intra‐individual stability of sleep structure include changes in lifestyle and occupational status (retirement), intervening life events, and somatic factors (Spiegel et al. 1999; Peters et al. 2014; Borbély et al. 2017).

While the decomposition of sleep into stages and other sleep parameters is useful to describe the actual state of sleep, the integration of these parameters may be more suitable for studying age‐dependent alterations and stable characteristics in sleep structure.

The present study has several limitations. The number of participants was rather small, and the original sample was composed of normal young subjects without any sleep complaints, mainly recruited from the academic community. This possibly reduced the variance of sleep parameters in Study 1.

Another limitation is that the present longitudinal study is based on a single follow‐up measurement after a very long interval, where no systematic and reliable information about intervening changes in sleep habits and life events is available. At the time of the original study, no follow‐up was planned. The single follow‐up measurement does not allow a detailed representation of the temporal course of sleep changes.

The small number of participants in the present study limits the statistical power to detect smaller and perhaps meaningful changes and to identify persistent characteristics of sleep over a very long period of time. Future longitudinal studies need to recruit a much larger prospective cohort with a broad spectrum of sleepers, with repeated recordings at shorter intervals of several years, and the analysis should include a wide range of parameters of sleep macrostructure and microstructure. Of course, this is a demanding long‐term task that requires a stable environment, and results cannot be expected in the near future. Some of the ongoing long‐term population studies, such as the Wisconsin Sleep Cohort (Young et al. 1993), the Sleep Heart Health Study (SHHS, Quan et al. 1997), the Hypnolaus study (Heinzer et al. 2011) and others have the potential to provide such data. The present study cannot be more than an encouragement for longitudinal sleep studies covering large parts of the life cycle.

## 5 Conclusions

The results of this longitudinal study, which spanned approximately 40 years, replicate the typical age‐related changes in sleep structure, including an increase in wake and stage 1 sleep, and a decrease in slow‐wave sleep and REM sleep. The present findings complement those of prior cross‐sectional studies. The absence of any significant correlation between the individual sleep parameters in the younger and the older age groups indicates that none of the examined single sleep parameters can be identified as a trait marker. Given the limited size of our sample, this does not exclude the possibility that in longitudinal studies with larger samples and a prospective design, trait characteristics of sleep macrostructure or microstructure could be identified. However, when a set of multiple sleep parameters was combined in a pattern analysis, it was revealed that the degree of concordance of the sleep pattern from young to older age depends critically on the absolute age of the participant, respectively on the age difference between the original and the follow‐up study. The degree of concordance between the findings of both studies decreased substantially in the age range from about 60 to 75 years. The results of the pattern analysis suggest that the individual sleep pattern may be more stable over time than the single sleep parameters. Consequently, the examination of a collective set of sleep parameters may prove to be an efficacious methodology for the identification of consistent sleep characteristics, which could potentially serve as sleep trait markers. To test this hypothesis, the most suitable method of investigation would be long‐term sleep studies in larger samples with repeated measurements.

## Author Contributions

**Peter Geisler:** conceptualization, methodology, writing – original draft, writing – review and editing, validation, supervision, funding acquisition, resources, investigation, visualization. **Renate Wehrle:** methodology, data curation, validation, formal analysis, project administration, writing – review and editing, visualization. **Alexander Yassouridis:** methodology, validation, formal analysis, visualization, data curation, writing – review and editing. **Alfred Ultsch:** methodology, formal analysis, visualization, writing – review and editing. **Thomas C. Wetter:** conceptualization, validation, supervision, funding acquisition, resources, writing – review and editing. **Hartmut Schulz:** conceptualization, methodology, investigation, validation, formal analysis, supervision, funding acquisition, writing – original draft, writing – review and editing.

## Ethics Statement

The study was approved by the ethics committee of the University Clinic of Regensburg (# 19–1557‐101, Oct. 09, 2019). Permission to reproduce material from other sources: n/a.

## Conflicts of Interest

The authors declare no conflicts of interest.

## Supporting information


Data S1.

## Data Availability

The data that support the findings of this study are available from the corresponding author upon reasonable request.
