# Supplementary material for: Sleep and Aging. A Polysomnographic Follow‐Up Study, Some 40 Years Later
Source: J Sleep Res. 2025 Mar 18;34(6):e70039. doi: 10.1111/jsr.70039 (PMC12592829; doi:10.1111/jsr.70039)
Supplement: Supplementary file 1 — Data S1. [file JSR-34-e70039-s001.docx]

Sleep and aging. A polysomnographic follow-up study, some 40 years later.

Supplemental material

Authors, Institutions

Peter Geisler^1^, Renate Wehrle^1^, Alexander Yassouridis^2^, Alfred Ultsch^3^, Thomas C. Wetter^1^, Hartmut Schulz^4^

1. Center of Sleep Medicine, Department of Psychiatry and Psychotherapy, University of Regensburg, Regensburg, Germany

2. Ethics committee, Ludwigs-Maximilians-University, Munich, Germany

3. Department of Informatics, Philipps-University, Marburg, Germany

4. Department of Education and Psychology, Free University, Berlin, Germany

Corresponding author:

Peter Geisler, Am Herrnberg 29, 93138 Lappersdorf, Germany

Email: peter.u.geisler@gmx.de

## Supplemental Material

**Table S1**

Questionnaire data of participants at Study 2

| Participant | **BDI** | **ESS** | **D-MEQ** | **PSQI** | **PSQI Q6** | **ISI** | **RIS** | **IRLS** |
| --- | --- | --- | --- | --- | --- | --- | --- | --- |
| SW01 | 9 | 5 | 61 | 3 | 0 | 5 | 5 | 3 |
| SW02 | 1 | 0 | 70 | 8 | 1 | 4 | 4 | 0 |
| SW03 | 6 | 9 | 62 | 7 | 1 | 0 | 6 | 0 |
| SW04 | 3 | 16 | 65 | 3 | 1 | 5 | 6 | 0 |
| SW05 | 0 | 11 | 51 | 3 | 1 | 4 | 5 | 0 |
| SW06 | 3 | 6 | 63 | 5 | 1 | 3 | 7 | 0 |
| SW07 | 4 | 6 | 30 | 8 | 2 | 7 | 14 | 0 |
| SW08 | 6 | 9 | 28 | 4 | 1 | 7 | 7 | 0 |
| SW09 | 0 | 5 | 53 | 3 | 1 | 0 | 4 | 0 |
| SW10 | 0 | 7 | 66 | 3 | 1 | 5 | 3 | 0 |
| SW11 | 6 | 2 | 54 | 4 | 1 | 3 | 10 | 0 |
| SW12 | 12 | 7 | 61 | 4 | 1 | 3 | 7 | 0 |
| SW13 | 0 | 3 | 54 | 1 | 0 | 2 | 5 | 1 |
| SW14 | 1 | 5 | 48 | 3 | 1 | 2 | 4 | 0 |
| SW15 | 4 | 6 | 57 | 4 | 1 | 3 | 3 | 13 |

Legend:

For all scales, except PSQI Question 6 (PSQI-Q6), the total score is given. For Study 1, no questionnaire data are available.

BDI = Beck’s Depression Inventory (Hautzinger et al., 1995)

ESS = Epworth Sleepiness Scale (Johns 1991)

D-MEQ = Horne-Oestberg Morning-Evening Questionnaire, German version (Griefahn et al., 2001)

PSQI = Pittsburgh Sleep Quality Index (Buysse et al. 1989)

PSQI-Q6 = PSQI, Question 6 (Overall sleep quality)

ISI = Insomnia Severity Index (Bastien et al., 2001)

RIS = Regensburg Insomnia Scale (Crönlein et al., 2013)

IRLS = International Restless Legs Syndrome Study Group Rating Scale (Trenkwalder et al., 2001)

For interpretation of scores see table S2

**Table S2**

Clinical interpretation of questionnaire scores and number of participants with respective score in Study 2

| **Scale / Score** | **Interpretation** | Participants with this score (N) |
| --- | --- | --- |
| **BDI** |  |  |
| 0–10 | No depression | 13 |
| 11-16 | Mild mood disturbance | 2 |
| 17-20 | Borderline clinical depression | 0 |
| 21-30 | Moderate depression | 0 |
| 31-63 | Severe to extreme depression | 0 |
| **ESS** |  |  |
| 0-10 | No excessive daytime sleepiness (EDS) | 13 |
| 11-16 | Mild to moderate EDS | 2 |
| 17-24 | severe EDS | 0 |
| **D-MEQ** |  |  |
| 16-30 | Definite evening type | 2 |
| 31-41 | Moderate evening type | 0 |
| 42-58 | Neutral circadian type | 6 |
| 59-69 | Moderate morning type | 5 |
| 70-86 | Definitive morning type | 1 |
| **PSQI** |  |  |
| <6 | Undisturbed sleep | 12 |
| ≥6 | Disturbed sleep | 3 |
| **PSQI-Q6** |  |  |
| 0 | Very good sleep quality | 2 |
| 1 | Fairly good sleep quality | 12 |
| 2 | Fairly bad sleep quality | 1 |
| 3 | Very bad sleep quality | 0 |
| **ISI** |  |  |
| 0-7 | No insomnia symptoms | 15 |
| 8-14 | subclinical insomnia symptoms | 0 |
| 15-21 | moderate insomnia symptoms | 0 |
| 22-28 | severe insomnia symptoms | 0 |
| **RIS** |  |  |
| 0-12 | No insomnia symptoms | 14 |
| >12 | Clinically relevant insomnia symptoms | 1 |
| **IRLS** |  |  |
| 0-10 | No or mild RLS | 14 |
| 11-20 | moderate RLS | 1 |
| 21-30 | severe RLS | 0 |
| 31-40 | very severe RLS | 0 |

Table legend

Ranges of questionnaire scores and related interpretation, according to validation studies. The number of participants who scored the respective values at study 2 is given in the right-hand column (n = 15). For citations of the scales see Table S1.

**Table S3:**

Comparison of Rechtschaffen & Kales and AASM stage scorings

|  | Rechtschaffen & Kales  Scoring | | AASM  Scoring | |
| --- | --- | --- | --- | --- |
|  | Mean | SD | Mean | SD |
| Wake % | 24.8 | 11.3 | 24.1 | 15.0 |
| S1 % | 10.5 | 4.0 | 9.8 | 4.4 |
| S2 % | 45.6 | 9.4 | 44.3 | 10.7 |
| SWS % | **2.8*** | **3.5** | **5.6*** | **5.4** |
| REM % | 16.4 | 4.1 | 16.2 | 5.6 |
| SOL (S1) (min) | 15.9 | 9.5 | 16.5 | 14.7 |
| SOL (S2) (min) | 19.6 | 11.4 | 19.6 | 16.3 |
| REM-latency (min) | 75.2 | 39.0 | 75.9 | 15.0 |

Table legend: Comparison of the scoring results according to the Rechtschaffen & Kales manual (1968) and the AASM guidelines (Iber et al., 2007) in the older age group (Study 2), mean values for three nights.

%: Percentage of the respective stage referenced to Time in Bed. SD: Standard deviation. All recordings were independently scored twice by two experienced scorers. Differences between R&K and AASM scoring were evaluated by paired Wilcoxon signed rank test (n = 15). *, bold typeface: significant difference, p < 0.001).

**Table S4**:

Sleep stage transition matrices

A) Young age group (Study 1)

| to🡺  🡻 from | Wake | S1 | S2 | SWS | REM | Total Transitions  (Epochs – 1) |
| --- | --- | --- | --- | --- | --- | --- |
|  | % | % | % | % | % | n |
| Wake | 84.8 | 13.4 | 1.1 | 0.1 | 0.6 | 2442 |
| S1 | **2.7** | 59.6 | 26.3 | 0 | **11.4** | 2279 |
| S2 | **0.5** | **1.3** | 90.3 | **6.5** | 1.4 | 19521 |
| SWS | 0.3 | 0.3 | **16.9** | **82.5** | 0.1 | 8108 |
| REM | **0.8** | 4.4 | 1.1 | 0 | 93.7 | 8858 |

B) Older age group (Study 2)

| to🡺  🡻from | Wake | S1 | S2 | SWS | REM | Total Transitions  (Epochs – 1) |
| --- | --- | --- | --- | --- | --- | --- |
|  | % | % | % | % | % | n |
| Wake | 82.1 | 15.7 | 0.9 | 0.1 | 1.2 | 10294 |
| S1 | **13.3** | 54.2 | 26.8 | 0.2 | **5.5** | 4388 |
| S2 | **2.9** | **2.8** | 90.8 | **2.6** | 0.9 | 19033 |
| SWS | 1.2 | 1.2 | **65.0** | **23.8** | 0 | 1184 |
| REM | **4.2** | 3.0 | 0.7 | 0 | 92.1 | 6858 |

**Table legend:** Stage transition matrices for the young (Panel A) and the older age group (Panel B). The originating stage of the transition is given in the rows, the target stage in the columns (e.g., in Study 2: transitions from Wake to S1 are 15.7% of all 10294 transitions originating from Wake). Shaded cells contain Intra-stage transitions. Higher percentages of intra-stage transitions correspond to more stable (contingent) episodes of consecutive epochs of the same sleep stage. Stages with intra-stage transitions > 50% are considered contingent. Total transitions: total number transitions originating from the stage in the respective row, equivalent to total number of 30-sec epochs of the stage in this row in 45 nights of the respective age group minus 1. Highlighted cells (bold) indicate significant differences between young and older age groups (paired Wilcoxon signed rank tests).

**Table S5:**

Number of completed REM periods and of REM cycles per night

|  | Number of completed REM-periods / night | | | | | | Mean number of completed REM periods / night ± SD | | Total number of completed REM periods in 3 nights | |
| --- | --- | --- | --- | --- | --- | --- | --- | --- | --- | --- |
|  | Y1 | Y2 | Y3 | O1 | O2 | O3 | Y1-3 | A1-3 | Young | Older |
| SW01 | 4 | 4 | 4 | 3 | 1 | 4 | 4.0 | 2.7 | 12 | 8 |
| SW02 | 2 | 4 | 4 | 3 | 4 | 4 | 3.3 | 3.7 | 10 | 11 |
| SW03 | 4 | 4 | 4 | 6 | 5 | 4 | 4.0 | 5.0 | 12 | 15 |
| SW04 | 2 | 3 | 3 | 4 | 5 | 5 | 2.7 | 4.7 | 8 | 14 |
| SW05 | 3 | 3 | 3 | 2 | 3 | 3 | 3.0 | 2.7 | 9 | 8 |
| SW06 | 4 | 3 | 5 | 0 | 4 | 3 | 4.0 | 2.3 | 12 | 7 |
| SW07 | 4 | 4 | 4 | 2 | 3 | 4 | 4.0 | 3.0 | 12 | 9 |
| SW08 | 3 | 4 | 3 | 3 | 2 | 2 | 3.3 | 2.3 | 10 | 7 |
| SW09 | 3 | 3 | 3 | 1 | 3 | 2 | 3.0 | 2.0 | 9 | 6 |
| SW10 | 3 | 3 | 3 | 4 | 3 | 4 | 3.0 | 3.7 | 9 | 11 |
| SW11 | 4 | 5 | 5 | 3 | 3 | 4 | 4.7 | 3.3 | 14 | 10 |
| SW12 | 5 | 4 | 2 | 5 | 5 | 5 | 3.7 | 5.0 | 11 | 15 |
| SW13 | 3 | 3 | 3 | 4 | 4 | 4 | 3.0 | 4.0 | 9 | 12 |
| SW14 | 4 | 4 | 4 | 4 | 3 | 3 | 4.0 | 3.3 | 12 | 10 |
| SW15 | 3 | 3 | 3 | 4 | 4 | 3 | 3.0 | 3.7 | 9 | 11 |
| **Mean/night** | **3.4** | **3.6** | **3.5** | **3.2** | **3.5** | **3.6** | **3.5 ±0.6** | **3.4±0.9** | **158** | **154** |

|  | Number of REM cycles / night | | | | | | Mean number of REM cycles / night ± SD | | Total number of REM cycles in 3 nights | |
| --- | --- | --- | --- | --- | --- | --- | --- | --- | --- | --- |
|  | Y1 | Y2 | Y3 | O1 | O2 | O3 | Y 1-3 | O 1-3 | Young | Older |
| SW01 | 3 | 4 | 4 | 2 | 1 | 3 | 3.7 | 2.0 | 11 | 6 |
| SW02 | 2 | 4 | 3 | 2 | 3 | 3 | 3.0 | 2.7 | 9 | 8 |
| SW03 | 4 | 4 | 4 | 6 | 5 | 3 | 4.0 | 4.7 | 12 | 14 |
| SW04 | 1 | 3 | 3 | 3 | 5 | 4 | 2.3 | 4.0 | 7 | 12 |
| SW05 | 3 | 3 | 2 | 2 | 2 | 3 | 2.7 | 2.3 | 8 | 7 |
| SW06 | 3 | 2 | 4 | 0 | 4 | 3 | 3.0 | 2.3 | 9 | 7 |
| SW07 | 4 | 4 | 4 | 2 | 3 | 3 | 4.0 | 2.7 | 12 | 8 |
| SW08 | 3 | 4 | 3 | 3 | 2 | 2 | 3.3 | 2.3 | 10 | 7 |
| SW09 | 3 | 3 | 3 | 1 | 2 | 2 | 3.0 | 1.7 | 9 | 5 |
| SW10 | 3 | 3 | 3 | 3 | 3 | 3 | 3.0 | 3.0 | 9 | 9 |
| SW11 | 4 | 5 | 4 | 3 | 3 | 4 | 4.3 | 3.3 | 13 | 10 |
| SW12 | 4 | 4 | 2 | 4 | 4 | 4 | 3.3 | 4.0 | 10 | 12 |
| SW13 | 3 | 2 | 3 | 4 | 3 | 3 | 2.7 | 3.3 | 8 | 10 |
| SW14 | 3 | 3 | 3 | 3 | 3 | 3 | 3.0 | 3.0 | 9 | 9 |
| SW15 | 3 | 3 | 3 | 3 | 3 | 3 | 3.0 | 3.0 | 9 | 9 |
| **Mean/night** | **3.1** | **3.4** | **3.2** | **2.7** | **3.1** | **3.1** | **3.2 ±0.5** | **3.0±0.8** | **145** | **133** |

Table legend: Number of complete REM periods and REM cycles per night. REM periods are considered incomplete when the sleep recording terminated within 20 minutes after the final REM epoch. The duration of a REM cycle was defined as the temporal distance between the first epoch of two consecutive REM periods. Y1, Y2, Y3: Night 1, 2, 3 in the young age group (Study 1); O1, O2, O3: Night 1, 2, 3 in the older age group (Study 2). Y 1-3, O 1-3: Mean value of nights 1 – 3 in the young and older age groups. Shaded columns refer to the older age group. **Table S6:**

REM Densities per REM period, by sequential position of the REM periods within the nights

| ID | REM1 | REM2 | REM3 | REM4 | REM5 | REM6 | Mean RD | SD |
| --- | --- | --- | --- | --- | --- | --- | --- | --- |
| SW01_Y | 1.29 (1) | 2.08 (2) | 2.39 (3) | 3.50 |  |  | 2.21 | 0.86 |
| SW01_O | 1.44 (1) | 2.91 (2) | 4.79 (3) |  |  |  | 2.83 | 1.90 |
| SW02_Y | 1.15 (1) | 1.46 (3) | 1.35 (2) | 1.99 |  |  | 1.44 | 0.51 |
| SW02_O | 3.64 (2) | 4.00 (3) | 3.21 (1) | 4.37 |  |  | 3.69 | 1.25 |
| SW03_Y | 1.44 (1) | 2.28 (2) | 2.61 (3) | 2.22 | 2.98 |  | 2.31 | 0.75 |
| SW03_O | 2.83 (1) | 3.37 (3) | 3.30 (2) | 3.54 | 3.59 | 2.39 | 3.22 | 1.16 |
| SW04_Y | 1.90 (1) | 1.93 (2) | 1.19 (3) |  |  |  | 1.81 | 0.53 |
| SW04_O | 2.28 (2) | 2.04 (1) | 4.13 (3) | 2.92 | 2.88 | 4.41 | 2.80 | 1.10 |
| SW05_Y | 1.97 (1) | 3.22 (2) | 3.48 (3) | 3.36 | 3.44 |  | 3.02 | 0.77 |
| SW05_O | 1.97 (1) | 3.44 (3) | 3.14 (2) | 3.79 |  |  | 2.94 | 1.07 |
| SW07_Y | 1.60 (2) | 1.43 (1) | 1.73 (3) | 1.58 | 1.73 |  | 1.61 | 0.53 |
| SW07_O | 1.94 (1) | 2.73 (3) | 2.21 (2) | 1.85 |  |  | 2.22 | 0.42 |
| SW08_Y | 2,00 (1) | 2.85 (2) | 3.00 (3) | 2.28 | 3.87 |  | 2.64 | 1.03 |
| SW08_O | 1.26 (1) | 2.94 (2) | 4.02 (3) | 4.39 |  |  | 2.91 | 1.49 |
| SW09_Y | 3.97 (2) | 5.07 (3) | 3.33 (1) | 3.69 |  |  | 4.16 | 0.87 |
| SW09_O | 3.58 (3) | 3.08 (2) | 2.5 (1) |  |  |  | 3.12 | 0.89 |
| SW10_Y | 4.15 (1) | 4.27 (3) | 4.17 (2) | 2.96 |  |  | 3.97 | 0.84 |
| SW10_O | 2.42 (2.5) | 2.00 (1) | 2.42 (2.5) | 2.51 |  |  | 2.34 | 0.69 |
| SW11_Y | 1.96 (1) | 3.01 (2) | 3.28 (3) | 2.89 | 4.48 | 4.92 | 3.24 | 1.34 |
| SW11_O | 0.88 (1) | 2.74 (2) | 3.20 (3) | 2.81 |  |  | 2.41 | 1.19 |
| SW12_Y | 1.69 (2) | 1.04 (1) | 2.86 (3) | 2.00 | 4.00 | 5.00 | 2.75 | 1.31 |
| SW12_O | 1.43 (1.5) | 1.43 (1.5) | 2.83 (2) | 3.15 | 3.7 |  | 2.51 | 1.14 |
| SW13_Y | 1.57 (1) | 2.62 (3) | 2.45 (2) | 2.85 |  |  | 2.33 | 0.85 |
| SW13_O | 2.16 (1) | 2.78 (3) | 2.35 (2) | 3.35 | 3.06 |  | 2.64 | 0.71 |
| SW14_Y | 2.06 (1) | 3.52 (3) | 3.44 (2) | 3.33 |  |  | 3.09 | 0.78 |
| SW14_O | 2.46 (1) | 3.22 (3) | 3.00 (2) | 3.17 |  |  | 2.96 | 0.48 |
| SW15_Y | 2.07 (1) | 2.62 (3) | 2.41 (2) | 2.10 |  |  | 2.3 | 0.52 |
| SW15_O | 2.23 (1) | 2.88 (2) | 3.66 (3) | 3.16 | 2.23 |  | 3.06 | 0.71 |
|  |  |  |  |  |  |  |  |  |
| Young |  |  |  |  |  |  |  |  |
| N | 42 | 42 | 39 | 32 | 28 |  |  |  |
| Mean | 2.09 | 2.77 | 2.75 | 2.57 | 3.02 |  |  |  |
| SD | 1.07 | 1.14 | 0.91 | 0.93 | 0.91 |  |  |  |
| Mean rank | 1.1 | 2.1 | 2.6 |  |  |  |  |  |
| Older |  |  |  |  |  |  |  |  |
| N | 42 | 42 | 40 | 28 | 10 |  |  |  |
| Mean | 2.18 | 2.83 | 3.18 | 3.02 | 3.46 |  |  |  |
| SD | 1.02 | 1.16 | 0.98 | 0.91 | 0.57 |  |  |  |
| Mean rank | 1.3 | 2.1 | 2.1 |  |  |  |  |  |

Table legend: Mean REM densities (RD) per REM sleep period per participant, across the three nights per study. SWxx_Y: data from Study 1 (young), SWxx_O: data from Study 2 (older age). REM1, REM2 … : Sequential position of the REM periods within the respective night. Values indicate RD in 3-second blocks per 30-second epoch of stage REM sleep (mean values for 3 nights). Numbers in brackets denominate the ranks (limited to the first three cycles for comparability) of RD in the respective nights, i.e., the REM period signed with (1) is the REM period with the highest mean value within the three nights in consideration. Shaded cells indicate values for the older age group. SW06: No data available for Study 1.
